# Biology of the Mealybug, *Phenacoccus solenopsis* on Cotton in the Laboratory

**DOI:** 10.1673/031.010.11501

**Published:** 2010-07-24

**Authors:** S Vennila, AJ Deshmukh, D Pinjarkar, M Agarwal, W Ramamurthy, S Joshi, KR Kranthi, OM Bambawale

**Affiliations:** ^1^ National Centre for Integrated Pest Management, LBS building, IARI, New Delhi, India; ^2^ Central Institute for Cotton Research, P.O.Box.2., Shankarnagar, Nagpur, Maharashtra, India; ^3^ lndian Agricultural Research Institute, Pusa campus, New Delhi, India

**Keywords:** crawlers, developmental period, parthenogenesis, fecundity, longevity

## Abstract

*Phenacoccus solenopsis* Tinsley (Hemiptera: Pseudococcidae) has been the current topic of research for insect taxonomists and applied entomologists in India due to its invasiveness, rapid spread, morphological and biological variations and the need for establishing an effective control strategy. The biology of the mealybug *P. solenopsis* was studied on cotton under laboratory conditions between August and October of 2009 with mean temperature and relative humidity of 23.3–30.2°C and 40.5–92.5% RH, respectively, in central India. Neonate crawlers that emerged from a field population were collected and constituted the study population. The developmental period from immature crawler to adult stage was greater for males (18.7 ± 0.9 days) compared to females (13.2 ± 1.8 days), probably due to the additional molt to the pupal stage in males. Survival of second instars was lower (45.5%) than first and third instars (71.4%). Females showed dynamic patterns of fecundity with the number of crawlers produced per female ranging between 128 and 812, with a mean of 344 ± 82. The reproductive period lasted 30.2 ± 8.2 days. Parthenogenesis with ovoviviparity (96.5%) was dominant over the oviparous (3.5%) mode of reproduction. Adult females lived 42.4 ± 5.7 days. Males accounted for less than 5% of the population, and lived 1.5 ± 0.1 days. The life history parameters of *P. solenopsis* adult females are discussed relative to the appearance of symptoms on the cotton crop, and the importance of making management interventions during the effective reproductive period of the insect.

## Introduction

The mealybug *Phenacoccus solenopsis* Tinsley (Hemiptera: Pseudococcidae) has a wide geographical distribution with its origin in Central America (Fuchs et al. 1991; Williams and Granara de Willink 1992) followed by reports of the Caribbean and Ecuador (Ben-Dov 1994), Chile (Larrain 2002), Argentina (Granara de Willink 2003), Brazil (Mark and Gullan 2005). *P. solenopsis* has been described as a serious and invasive pest of cotton in Pakistan and India (Hodgson et al. 2008) and on *Hibiscus rosa-sinensis* in Nigeria (Akintola and Ande 2008). Latest report on the invasiveness of *P. solenopsis* has been from the Eastern region of Sri Lanka (Prishanthini and Laxmi 2009) on ornamentals, vegetable crops, and weeds, and in China (Wang et al. 2009; Wu and Zhang 2009) on cotton.

In India, occurrence, severity, and epidemic forecast of mealybugs on cotton were made at Gujarat during the 2004–05, 2005–06, and 2006–07 crop seasons. The identity of the species involved was documented as *P. solenopsis* by Jhala and Bharpoda (2008a) and Jhala et al. (2008). However, Bambawale (2008a and b) reported the occurrence of *P. solenopsis* a decade ago from non-cotton growing areas of Uttar Pradesh, Madhya Pradesh, and Karnataka states of India and described it as a non-invasive pest. A detailed comparative study of few species of *Phenacoccus* including the Indian and Pakistan species, and details on the existence of seasonal morphological variations in *P. solenopsis* by Hodgson et al. (2008) provided strong support for its presence in India. However, a recent paper by Abbas et al. (2009) describes the dominant mealy bug species of Pakistan as *P. gossypiphilous.* Widespread infestation of *P. solenopsis* and economic damage to cotton across nine cotton growing states of the country including Punjab, Haryana, Rajasthan, Gujarat, Madhya Pradesh, Maharashtra, Andhra Pradesh, Karnataka, and Tamil Nadu during 2008–09 crop season led to a national level summit at the Central Institute for Cotton Research (CICR) formulated strategies for its management (Dharajyoti et al. 2008; Dhawan et al. 2008 and 2009; Jhala and Bharpoda; 2008 b, c; Suresh and Kavitha 2008 a, b). A survey across 47 locations between late 2007 and early 2008 established the predominance of *P. solenopsis* (Nagrare et al. 2009) in India. The present report investigates the biology of *P. solenopsis* under laboratory conditions at Nagpur in the rain-fed cotton growing central zone of the country.

## Materials and Methods

Studies on biology of *P. solenopsis* were carried out in the laboratory at CICR, Nagpur using the population collected from unsprayed cotton fields of *Gossypium hirsutum* L. (Malvales: Malvaceae) at the experimental station. Twigs of cotton plants infested with reproducing females of *P. solenopsis* were brought to the laboratory; individual females were separated, and fed on cotton leaves in Petri plates. Specimens of mealybugs (registered under RRS No. 2132-34/08) used in biology studies were confirmed to be *P. solenopsis* by the Insect Identification Service of Indian Agricultural Research Institute, New Delhi. Individual cotton leaves with petioles collected from fifth position of the plant terminal of the *G. hirsutum* Bt hybrid, grown in net houses without insecticidal spray and free from mealybug infestation, were washed with tap water and shade dried and used as food source. Since parthenogenetic reproduction of *P. solenopsis* was observed under field conditions, individual neonate crawlers emerging from females were used as to start the biology study. The base of the petiole of individual leaves were covered with a water soaked cotton swab to prevent desiccation of the leaf. A total of 250 crawlers drawn from different females but laid on the same day were individually transferred to separate glass Petri plates (15 × 2 cm) each containing a cotton leaf. The study was conducted between August and October 2008 in the laboratory when maximum and minimum temperature and mean relative humidity of the study area ranged from 25.6 to 36.5°C and 14.2 to 25.5°C, and 40.5 to 92.5% RH respectively.

Observations on survival and molt of the crawlers were recorded daily under stereoscopic microscope until they became adults. Unless the crawlers were in a pre-molt stage, Petri plates along with cotton leaves were changed on alternate days or else transferred after the molt. Transferring the cut cotton leaf disc along with crawler obviated their direct handling using a camel hair brush (No.1). Petri plates with missing crawlers were discarded and excluded from the final data. The developmental time of each instar was recorded based on an observed exuvia. Daily monitoring of crawlers, those that had stopped further molting and reached adult stage was done to determine the prereproductive and reproductive periods, fecundity, and longevity. As the eggs or neonate crawlers were counted and discarded, the individual adults that had produced them were transferred to new Petri plates for further observations. When eggs were observed they were separated along with the leaf disc and observed until they hatched.

Range and mean values for the developmental period of each instar for females and males, pre- reproductive and reproductive periods, fecundity, and longevity were calculated for each life stage based on the total number of observations made. The number of observations for each of the life history parameter varied depending upon the progress in development and survival of the crawlers and adults. The number of males out of the total population that survived to adult stage was calculated. Since the reproductive period was longer and the adults had fecundity varying from one to many (>200) crawlers per day, the criteria of a minimum number of 10 crawlers produced per day was considered to determine the period of effective reproduction by females.

## Results

The developmental period of crawlers of *P. solenopsis* was shorter and similar for first and third instars (2–6 days), and longer for the second instar (2–11 days). Mean developmental periods of first, second and third instars were 3.9 ± 0.4, 5.1 ± 3.2 and 4.2 ± 0.6, respectively. Males had an additional instar and prepupal stage over 5–7 days of development with a mean of 5.5 ± 0.5 days. The mean total developmental period for crawlers with three instars, and four instars that developed into females and males, was 13.2 ± 1.8 and 18.7 ± 0.9, respectively. Females had a wider range of developmental periods than males. While the survival of first and third instars was the same (71.4%), the second instar had only 45.5% survival, and females survived (92.7%) better than males (83.3%). (Table 1). Females after the final moult took about 2–8 days for reproduction with a mean pre reproductive period of 5.7 ± 1.7 days. Reproduction by *P. solenopsis* was parthenogenetic with 96.5 and 3.5% of offspring produced as crawlers and eggs through ovoviviparity and oviparity, respectively. Under laboratory conditions the typical occurrence of an ovisac was missing although neonates or eggs were entangled in hyaline waxy thread-like structures. Mean fecundity was 334.4 ± 82 with a range of 128– 812 crawlers per female. The maximum incubation period of eggs was 120 min with a mean of 68.5 ± 33.0 min. The duration of reproduction was as short as 10 days to a maximum of 47 days with a mean of 30.2 ± 8.2 days. Offspring production by adult females was disjunctive with one to seven non-reproductive periods with a mean of 2.4 ± 0.6 days interspersed between the reproductive phase of the life cycle. More than 10 crawlers per day were produced by females for a minimum and maximum period of 6 and 30 days,, with a mean effective reproductive period of 17.2 ± 4.3 days, during which 97.3% of crawlers were produced. Mean longevity of adult females was 42.4 ± 5.7 days with a range of 36–51 days (Table 2). Adult females at the end of reproduction died the very next day or lived up to a maximum of 6 days. Males were winged, delicate and nonfeeding. The proportion of males to the total population used in the study was 0.05, and they lived for a maximum of 2 days with a mean of 1.5 ± 0.1 days. During the study period, temperature and relative humidity conditions of the laboratory depended on outdoor weather variations of the study area as the laboratory was a screened enclosure. The monthly mean of maximum and minimum temperatures, and relative humidity were: August (30.8, 31.3 and 32.1°C and 77.7%), September (24.3, 24.0 and 22.8°C and 76.9%) and October (24.3, 24.0 and 22.8° C and 71.1%).

**Table 1.**
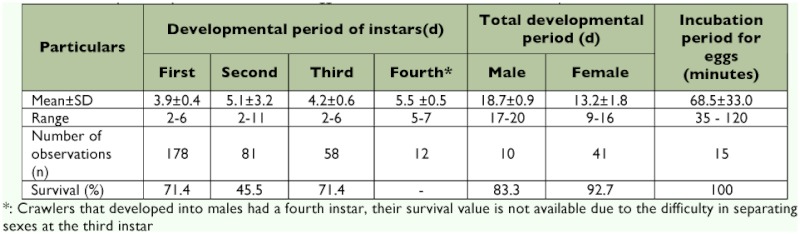
Developmental period and survival of eggs and crawlers of *Phenococcus solenopsis*

## Discussion

To understand the mode and degree of its population growth of an insect pest, it is important the to understand the environmental conditions of the crop. Although the reports of occurrence and epidemics of *P. solenopsis* have been documented on cotton from several countries (Jhala and Bharpoda 2008b; Wang et al. 2009), details of the biological parameters were not explored due to the need for extensive standardization of culture materials and methods. Since a study of the life history and pattern of biological activities are difficult under field conditions of cotton because of the interference of biotic and abiotic factors, laboratory studies have become essential. Studies conducted in the laboratory using cotton leaves placed in Petri plates with detailed observations of reproductive and developmental stages of *P. solenopsis* formed the basis for the present study. Cotton leaves collected from the same position on the plant from only one cultivar provided a similar food source for developing mealybugs thus avoiding any variation in food quality. Since individual leaves could be placed in Petri plates, they were easily observed under the microscope.

**Table 2.**
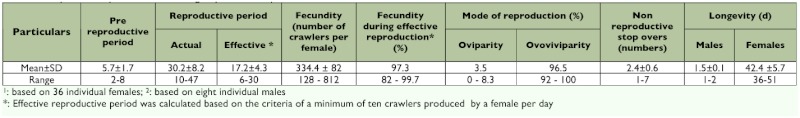
Reproductive parameters1 and longevity2 of *P. solenopsis*

Longer developmental duration of males compared to females was due to an additional molting and prepupal processes. While the longer developmental period of the 2^nd^ instar of males along with their high mobility could be the reason for their lower survival, it was not observed in the fourth instar due to the scarce population of males, together with the difficulty of observation of any sex related differences during early crawler stages. Akintola and Ande (2008) studied *P. solenopsis* on *H. rosa-sinensis* and found progressively increasing developmental periods of 6, 8 and 10 days for the 1st, 2nd and 3rd instars, respectively. However *P. solenopsis* under laboratory conditions had longer developmental periods for the 2nd instar over the other two instars, indicating the influence of ecological zone with the associated weather conditions as well as host plants that could influence *P. solenopsis* development. The total developmental duration of a closely related species *Phenacoccus madeirensis* reared under constant temperatures of 25, 20 and 15°C was reported to be 30, 46 and 66 days respectively (Chong et al. 2003). Much shorter developmental periods of *P. solenopsis* together with the pattern of crawler production and wider range of fecundity by females observed in the present study under more variable day to day temperature and humidity conditions suggested that *P. solenopsis* has become acclimatized to a tropical environment that may have allowed its rapid spread across widely differing agro climatic zones of the Indian continent. Lower numbers and shorter life span of males suggested that they have a minor role in reproduction, although under field conditions sexual reproduction also could be a possibility.

Viewed in conjunction with the biology of *P. solenopsis* it is quite clear that the longevity of the adults, and their larger size with increased waxy coating, and higher food requirement, result in visibility of the pest and symptoms on the crop. Therefore, with the initial notice of *P. solenopsis* infestation on few plants it is essential to monitor the plants regularly for at least 14 to 20 days, which is when reproduction by females occurs, to make management decisons for using insecticidal sprays. Higher mortality of the crawlers, the longer effective reproductive period and increased longevity of adult females along with the expected natural mortality factors such as predation, parasitization and action of abiotic factors on crawlers and adults under natural field conditions, suggest that management interventions should be focused against reproducing adult females rather than crawlers to prevent the multiplication and spread of the pest. Therefore bioassay studies should use adult females instead of crawlers to determine an efficacious management scheme.

Having established the species identity of *P. solenopsis* and its invasive nature into Pakistan and India, the parthenogenic type of reproduction by *P. solenopsis* observed in the present study is different from description of reproduction as sexual by Hodgson et al. (2008). Given the results of the present report, it appears that *P. solenopsis* is variable in terms of behavioral and developmental patterns. Tanwar et al. (2007) described many species of mealybugs, including *P. solenopsis*, and attributed the buildup of mealy bugs to abiotic changes in the environment. The narrow genetic diversity of *P. solenopsis* population established across the country by molecular studies (ICAC recorder 2008) point out the decisive role of ecological influences on the biology of *P. solenopsis.* Studies that are under way to determine developmental rates at different constant temperatures in growth chambers, ability of *P. solenopsis* to multiply, survive and spread across regions among many host plants, and the continuing molecular studies on the variations in their populations would be able to resolve and strengthen the species identity, biology and effect of environmental factors.
